# The use of linked administrative data to explore the impact of formal social care services involvement in childhood on education and offending outcomes.

**DOI:** 10.23889/ijpds.v7i3.1902

**Published:** 2022-08-25

**Authors:** Anna Leyland

**Affiliations:** 1 University of Sheffield

Young people who are care experienced are overrepresented in the criminal justice system and in general have worse outcomes in education than their non-looked after peers (DfE & Hinds, 2019; Prison Reform Trust, 2017). Recent evidence has reported that children who have a social worker (classified as child in need or those with a child protection plan), but who do not enter the care system, can have worse outcomes in education than those children who are looked after (DfE, 2019b). There remains little research evidence about the extent to which children with a social worker become involved with criminal justice services or how the age, duration and nature of formal social care service involvement may impact on education and offending outcomes. Researchers have been challenged by the lack of a sufficiently sized quantitative data, that crossed social care, education and offending domains. In work funded by ADR-UK, the present research project uses the newly linked DfE-MoJ administrative dataset to explore how different forms of formal social care services involvement impacts on education and criminal justice outcomes. Multi-level models will explore the role of formal child social care services amongst other multi-systemic risk and protective factors for education and offending. The presentation of his work will include preliminary statistics and insights into the challenges and potential further use of the linked dataset for researchers.

